# Coordination of a Dual-Channel Pharmaceutical Supply Chain Based on the Susceptible-Infected-Susceptible Epidemic Model

**DOI:** 10.3390/ijerph17093292

**Published:** 2020-05-08

**Authors:** Yanhong Hou, Fan Wang, Zhitong Chen, Victor Shi

**Affiliations:** 1 School of International Pharmaceutical Business, China Pharmaceutical University, Nanjing 211198, China; 1020092084@cpu.edu.cn; 2 Human Resources Department, Guizhou University, Guiyang 550025, China; fwang5@gzu.edu.cn; 3 School of Physical Education and Health, Shanghai Lixin University of Accounting and Finance, Shanghai 201209, China; 4 Lazaridis School of Business and Economics, Wilfrid Laurier University, Waterloo, ON N2L 3C5, Canada; cshi@wlu.ca

**Keywords:** susceptible-infected-susceptible epidemic model, pharmaceutical supply chain, dual channels, supply chain coordination

## Abstract

With the continuous development of Internet, online pharmaceutical channels in many countries have seen rapid expansion. As a result, pharmaceutical supply chain participants can adopt dual channels, namely, both online channels and offline channels. As online channels compete with traditional offline channels, it is of great relevance to study the potential conflicts and coordination between them, which is the focus of this paper. Specifically, this article develops a susceptible-infected-susceptible epidemic model of the dual channels for a pharmaceutical supply chain. Our main findings are that in a competitive situation, there is a positive stable equilibrium. Furthermore, increasing the rate of influence of offline transmission, online transmission, and cross transmission will improve sales. Moreover, improving the transmission influence rate will turn more potential customers into purchasers, increase channel sales, and achieve dual channel coordination. We then conduct numerical analysis to illustrate and complement the findings from the model. Finally, we provide managerial insights for implementing successful dual-channel pharmaceutical supply chains.

## 1. Introduction

With the continuous development of the Internet, online pharmaceutical channels in many countries have expanded rapidly. The United States was one of the first countries to initiate pharmaceutical sales on the Internet. As early as 1991, the United States launched the online pharmacy website certification program and became the pioneering country in online pharmaceuticals. This has led to the current prevalent coexistence of business to business (B2B), business to consumer (B2C), and third-party pharmaceutical Internet trading platforms in the United States. Among them, B2B mode is the most popular one, where suppliers, hospitals, pharmacies, and clinics are integrated. B2C services such as online diagnosis and treatment and online pharmacies also has a large market share. At present, there are more than 1000 online pharmacies and more than 20,000 health-related websites in the United States, and online channels of pharmaceutical sales have become the main growth point of e-commerce in the United States. Furthermore, the UK, Germany, Italy, Switzerland, Japan, and many other countries have a large proportion of online trading. In this paper, we define online channels and offline channels together as dual channels, which means that pharmaceutical supply chain participants can adopt both online channels and offline channels. 

Pharmaceutical supply chains are socio-technical systems designed to align firms in order to enable improved health [1]. When a pharmaceutical supply chain involves both online and offline channels, its participants face conflicts in choosing the channel. Channel conflicts can be divided into internal conflicts and external conflicts. Internal conflicts are the contradiction between different departments of the same enterprise. External conflicts refer to the contradiction between two or more independent enterprises. External conflicts can be divided into horizontal conflicts, vertical conflicts, and dual-channel conflicts. Horizontal conflicts are the conflicts between different enterprises at the same level, such as channel conflicts between distributors in different pharmaceutical supply chains. Vertical conflicts are the conflicts between different levels of enterprises in a supply chain, which are more common than horizontal conflicts. For example, the conflicts between distributors and manufacturers, and the conflicts between distributors and retailers. Dual-channel conflicts are the conflicts between different channels, such as online and offline channels. Channel conflicts have both positive and negative effects. Multiple channels may lead to cross-selling, competition for customers and resources, and channel conflicts. As online channels compete with the traditional offline channels, it is therefore of great significance to study the conflicts between online and offline channels. 

A number of existing studies focus on dual-channel price coordination problems [2,3,4]. Some studies use supply chain contracts to coordinate the dual-channel [5,6,7], assuming two-stage supply chain distribution systems. For example, reference [8] discussed a manufacturer with two channels, based on the theory of consumer utility. The authors found that the demand of each channel was only affected by prices. Reference [9] considered coordination from three potential perspectives: manufacturer Stackelberg, retailer Stackelberg and Nash equilibrium. The authors showed that price discount could improve the profits of the supply chain members. Reference [10] also employed game theory to study a manufacturer’ dual-channel pricing strategy and showed that a single wholesale price contract could not coordinate channels. However, changing the retail price within a certain range could achieve coordination. References [11] and [12] reviewed the research on pharmaceutical supply chain management and argued that related research on dual channel is rare. Reference [13] pointed out that drug users increasingly purchase drugs online, and purity and adulteration do not vary considerably between drugs purchased online and offline for most substances, while online prices are mostly higher than offline prices. Reference [14] presented a model of multichannel distribution between two separate companies, particularly in the area of retail organizations. 

Moreover, scholars have studied the stability of markets and channels borrowing the knowledge from the field of Biology to study dual-channel coordination problem. Reference [15] explored the origins of different channel and investigated the efficiency and evolution of the channel change under the generalized Lotka–Volterra model. Reference [16] used a logistic equation accurately describing the growth law of populations of the channel. The authors also studies population sustainability of a channel [17,18]. Reference [19] studied the self-governing susceptible-infected-susceptible (SIS) epidemic model of two competing channels. In [20], a dual- channel competition model was built and the evolution and stability of consumers’ purchasing behavior in different channels was discussed. Those studies established the foundation for the SIS model for pharmaceutical supply chains and for stability analysis and how to derive the population at different channels.

The rest of this paper is organized as follows. In Section 2, a dual-channel pharmaceutical supply chain structure consisting of an online channel and an offline channel is discussed, and the main mode of each channel is introduced. Section 3 describes the construction of the SIS model of the dual channel for the pharmaceutical supply chain. Section 4 discusses the two-dimensional and four-dimensional equilibrium points and the stability of equilibrium points in the SIS model. Finally, the paper concludes with simulation analyses and discussions. 

## 2. Dual-Channel Pharmaceutical Supply Chain Structure

In the case of pharmaceuticals, the dual-channel system has both positive and negative effects. The offline channel of the pharmaceutical supply chain includes pharmaceutical manufacturers, distributors, retailers, doctors, and patients. The online channel also comprises trading platforms as channel members, including self-operated platforms and third-party platforms. Different countries have different regulations on pharmaceutical distribution. For example, online B2B pharmaceutical transactions is very common among Italian pharmaceutical enterprises, but online B2C transactions are prohibited by regulations. Pharmacies are the main sales channels in the United States, France, Germany, and some other countries, while China sells pharmaceuticals to patients mainly through hospitals. Table 1 and Table 2 summarize various offline and online pharmaceutical supply chain structures.

In this context, although offline and online channels together expand the scope of consumers and increase market share, they present various advantages and disadvantages to each member of the supply chain. Online channels help pharmaceutical manufacturers obtain more profits, but simultaneously, the profits of distributors decrease, because of direct online transactions between the manufacturers and downstream retailers. As far as pharmaceutical retailers are concerned, on the one hand, online channels increase the profits of enterprises, but on the other, offline channels sales transfer to online channels, thereby damaging the profits of offline channels. 

Although online channels cannot provide a face-to-face professional service, with the development of electronic payment and logistics, they facilitate one-stop purchasing for buyers. As professional consultants are also available online, consumers can purchase directly through online channels. In terms of marketing, online channels pay more attention to the maintenance of the website, the choice of online products, the improvement of logistics services, etc. Online channels thus reduce on-site marketing costs. For B2B, the online channel can not only make available information on demand, supply, transfer, and bidding related to pharmaceuticals, but consumers can also compare pharmaceuticals prices, place orders, and so on. Logistics information can be shared among manufacturers, distributors, retailers, and consumers. Manufacturers, distributors, and retailers can also rationally plan their manufacturing and inventory accordingly. 

In the traditional offline channels, consumers obtain pharmaceuticals information mainly by the introduction of pharmaceuticals packaging and through pharmacists. The purchase choice is also based on the brand effect or the recommendation of the pharmacist. Contrastingly, for consumers on online B2C channels, a simple click on the web page can provide information on pharmaceuticals, such as brands, prices, and detailed assessments. Consumers on online channels can thus make the most economical and effective purchase choices based on this information. As a result, competition between offline and online channels may lead to price chaos and cost increases, and it is therefore essential to understand the competition between the two channels.

Therefore, in this study, we use a dual-population infectious disease model to simulate the sales process of identical or similar products sold by a pharmaceutical supply chain using offline and online channels simultaneously.

## 3. The Susceptible-Infected-Susceptible Epidemic Model for Pharmaceutical Dual-Channel Supply Chain

The susceptible-infected-susceptible (SIS) epidemic model, sometimes also called the infectious disease model or the SIS model, is a mathematical model to study the process of transmission of infectious diseases. The infectious disease model has been widely accepted after more than a century of development. It was initially used to study the impacts of diseases [21,22], and was then gradually applied to various other fields such as risk transfer [23], rumor spreading [24], and information spreading [25].

The decision-making process of consumers in the dual channels of the pharmaceutical supply chain is very similar to the transmission of infectious diseases. The susceptible people are those who are likely to be infected: “infected people”, refers to the customers who have already purchased the pharmaceutical’s products through the dual channels; the cured people are those who have recovered through treatment and will be removed from the infected people group. They may acquire immune capacity and no longer buy pharmaceutical products. In this paper, the infectious disease model is used to study consumers’ decision-making behavior and to explore the interaction between the two channels, so as to provide suggestions for the dual-channel development of the pharmaceutical supply chain.

### 3.1. The Infectious Disease Model in a Dual-Channel Pharmaceutical Supply Chain

#### 3.1.1. The Consumer Purchase Process

(1) Demand formation. There are two kinds of consumers: individual and corporate. Individual consumers purchase pharmaceutical products when they suffer from a disease or have some health-care need; corporate consumers may purchase pharmaceuticals when it is out of stock or inventory falls to the reorder point.

(2) Understanding and comparing pharmaceuticals. To make the most reasonable and effective purchase decision, consumers need to know the function, price, and other information in advance to choose and purchase pharmaceutical products. The purchase criteria can be a measure of the effectiveness of each potential product for the consumer. It can also represent the utility for the customer from purchasing a pharmaceutical candidate from each potential channel.

(3) Deciding on a purchase choice and consumers form purchases. Through pharmaceuticals comparison, consumers choose a pharmaceutical product and buy it from one of the channels. If there is no urgent need for the product or if it does not meet the requirements, the consumers choose not to buy it.

(4) Purchase or repeat purchase. Individual consumers terminate purchasing behavior because their disease is cured or they repeat the purchase because the disease is not cured; corporate consumers stop purchasing because the pharmaceutical sales are lower than expected, or they may re-purchase because of good sales.

For clarity, the customers who form purchase demand, compare pharmaceuticals, or decide on a purchase choice are referred to as potential customers. The consumers who form a purchase behavior are referred to as purchased customers. Finally, the consumers who no longer need to purchase are referred to as cured customers.

#### 3.1.2. Comparison between the Infectious Disease Model and the Dual-Channel Model of Pharmaceutical Supply Chain

**Figure 1:**
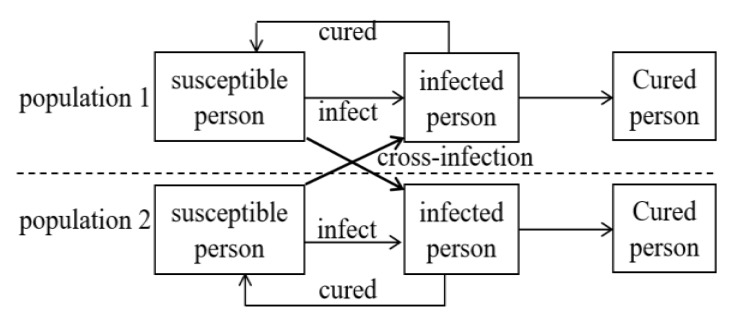
The transmission process of infectious diseases between two populations.

**Figure 2:**
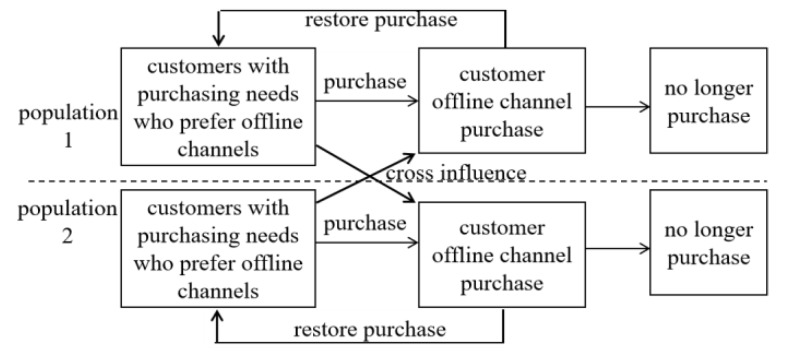
The customer’s purchase process in the pharmaceutical dual-channel supply chain.

As shown in Figure 1, in the two-population infectious disease model, the infectious diseases exist in both populations. There are three different groups in each population: the susceptible, the infected, and the cured. There is a cross-infection phenomenon, which means an infected person in the population 1 can infect a susceptible person in the population 2, or vice versa. In the dual-channel system of pharmaceuticals enterprises, as shown in Figure 2, the online and offline channels are similar to the two populations in the transmission process of infectious diseases. Purchasing the same pharmaceutical is similar to spreading infectious diseases in the two populations. There are three relationships among populations:

1. Competition, which refers to the phenomenon that two populations living in the same environment compete for ecological resources;
2. Mutually beneficial symbiosis, which refers to the mutual benefit of two populations and when they are separated, neither of them can live independently;
3. Parasitism, which refers to the fact that two populations benefit from and suffer due to each other. They become parasitic and host populations, respectively.

In the infectious disease model, a susceptible person is someone who has not been infected but is more likely to be infected. This corresponds to a potential consumer who has not yet purchased pharmaceuticals from the dual channels. An infected person is someone who has been infected with the disease, corresponding to a customer who has already purchased the pharmaceutical in the pharmaceutical supply chain. A cured person is someone who has recovered through treatment. S/he may stop buying because of acquiring immunity, or s/he may buy again because of reinfection.

### 3.2. Models and Assumptions

**Figure 3:**
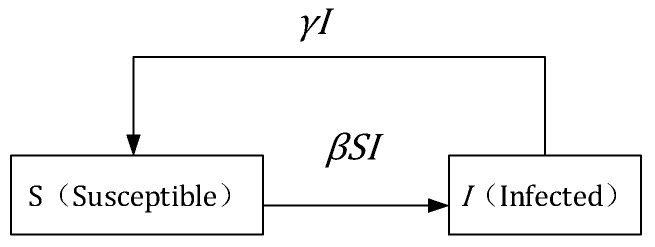
Single channel susceptible-infected-susceptible (SIS) infectious disease model.

When people are infected with a disease, such as meningitis, gonorrhea, etc., they can recover and do not acquire immunity after rehabilitation. The single-channel SIS infectious disease model is shown in Figure 3.

In the single channel SIS model, the population is a closed loop, regardless of birth or death. Hence, N=S(t)+I(t) is a constant. γI indicates the number of recoveries and γ indicates recovery rate of infected persons. In the SIS model, the following differential equation is satisfied:

(1) $$\left\{ \begin{array}{l} \frac{dS}{dt}=-\beta SI+\gamma I\\ \frac{dI}{dt}=\beta SI-\gamma I \end{array}\right.$$

To simplify the model, we make the following assumptions:

1. The pharmaceutical products sold in the online and offline channels are similar or identical;
2. At a given time, a consumer either has purchased a product or has demand for it;
3. At a given time, a consumer only buys pharmaceuticals from one channel;
4. At a given time, consumers’ preferences for one buying channel can change to the other; that is, online channel consumers can transform to the offline channel, or vice versa.

**Figure 4:**
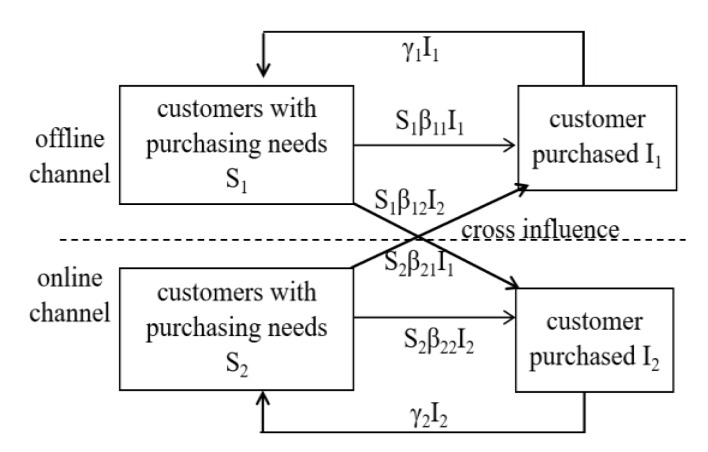
The dual-channel infectious disease SIS model for the pharmaceutical supply chain.

As shown in Figure 4, a dual-channel infectious disease SIS model for pharmaceutical supply chain is established. Unlike a single-channel SIS infectious disease model, dual-channel models have mutual influence and transformation in offline and online channels.

### 3.3. Results

In the dual channels, for rational consumers, the sales of one channel will affect the sales volume of the other channel. Therefore, the two channels are generally in a competitive and substitutable relationship. If an enterprise wants to maximize the total profit or revenue from the two channels, they must coordinate. In this paper, the SIS model is adopted to study the channel stability, and the coordination between the online and offline channels. We have the following two propositions:

**Proposition** **1.** *Only in the competitive situation, there is a stable equilibrium point.*

**Proposition** **2.** *The positive impact of the purchased customers on the potential customers increases the sales volume.*

### 3.4. The Dual-Channel Infectious Disease Sis Model 

Based on the SIS epidemic model proposed by [19], we construct the competitive dual-channel infectious disease SIS model for the pharmaceutical compound channel, as shown in the Equation (A1).

## 4. Model Solution

### 4.1. Equilibrium Point

We solve (Equation (A1)) and predict the purchase situation of customers in the dual channels of pharmaceutical companies based on the equilibrium point. In this way, we can judge the trend and stability of the two channels. Since *N_i_*, *S_I_*, *I_i_* are linearly related, as well as *I* and *I_i_*, and the main parameter is the amount of customer purchase, the system can be simplified into a four-dimensional system of (*N*_1_, *I*_1_, *N*_2_, *I*_2_), show in the Equation (A2).

#### 4.1.1. Two-Dimensional Equilibrium Point

*N*_1_ and *N*_2_ are relatively independent. Thus, we can form a two-dimensional system for *N*_1_ and *N*_2_, shown in Equation (A3). We can then obtain the equilibrium points $O{\left(0,0\right)}^{T}$, $P{\left(K_{1},0\right)}^{T}$ and $Q{\left(0,K_{2}\right)}^{T}$, which are in area *G*: $G=\left\{{\left(N_{1},N_{2}\right)}^{T}|K_{i}\geq N_{i}\geq 0,\;i=1,2\right\}$. Positive equilibrium points $M{\left(N_{1E},N_{2E}\right)}^{T}$, ${\left(N_{1E},N_{2E}\right)}^{T}$ are the positive solution of Equation (A4), shown in Appendix A.

We have $N_{1E}=\frac{\left(m+r_{1}\right)r_{2}K_{1}}{r_{1}r_{2}-mn}$, $N_{2E}=\frac{\left(n+r_{2}\right)r_{1}K_{2}}{r_{1}r_{2}-mn}$, and $r_{1}r_{2}-mn\neq 0$, $\left(m+r_{1}\right)r_{2}K_{1}\neq 0$, $\left(n+r_{2}\right)r_{1}K_{2}\neq 0$. Since $K_{i}\geq N_{i}\geq 0,i=1,2$, we have $K_{1}\geq N_{1}\geq 0$, $K_{2}\geq N_{2}\geq 0$, then $K_{1}\geq \frac{\left(m+r_{1}\right)r_{2}K_{1}}{r_{1}r_{2}-mn}\geq 0$, $K_{2}\geq \frac{\left(n+r_{2}\right)r_{1}K_{2}}{r_{1}r_{2}-mn}\geq 0$, that is $1\geq \frac{r_{1}r_{2}+mr_{2}}{r_{1}r_{2}-mn}\geq 0$, $1\geq \frac{r_{1}r_{2}+nr_{1}}{r_{1}r_{2}-mn}\geq 0$.

The existence conditions of the positive equilibrium point *M* are:

1. When $r_{1}r_{2}-mn>0$, that is $r_{1}r_{2}>mn$, then $mr_{2}\leq -mn,\;nr_{1}\leq -mn,\;\left(m+r_{1}\right)r_{2}K_{1}>0$, $\left(n+r_{2}\right)r_{1}K_{2}>0$. At this point, $r_{1}>-m,\;r_{2}>-n$; Therefore, *m*, *n* are negative, and $r_{1}>-m,\;r_{2}>-n$.
2. When $r_{1}r_{2}-mn<0$, that is $r_{1}r_{2}<mn$, then $r_{1}<-m,\;r_{2}<-n$, where *m* and *n* are negative.

The influence rate between the two channels is negative, indicating that there will be a positive equilibrium point when the channels are in competition. Based on the above analysis, we further obtained the following results:

1. When $\left\{ \begin{array}{l} r_{1}r_{2}>mn\\ r_{1}>-m\\ r_{2}>-n \end{array}\right.$ or $\left\{ \begin{array}{l} r_{1}r_{2}<mn\\ r_{1}<-m\\ r_{2}<-n \end{array}\right.$, m<0 and n<0. The two-dimensional system has four equilibrium points in Area *G*: $O{\left(0,0\right)}^{T}$, $P{\left(K_{1},0\right)}^{T}$, $Q{\left(0,K_{2}\right)}^{T}$ and $M{\left(N_{1E},N_{2E}\right)}^{T}$.
2. When $\left\{ \begin{array}{l} r_{1}r_{2}>mn\\ r_{1}<-m\;\mathrm{or}\;r_{2}<-n \end{array}\right.$ or $\left\{ \begin{array}{l} r_{1}r_{2}<mn\\ r_{1}>-m\;\mathrm{or}\;r_{2}>-n \end{array}\right.$, the two-dimensional system has three equilibrium points in Area *G*: $O{\left(0,0\right)}^{T}$, $P{\left(K_{1},0\right)}^{T}$ and $Q{\left(0,K_{2}\right)}^{T}$.

**Figure 5:**
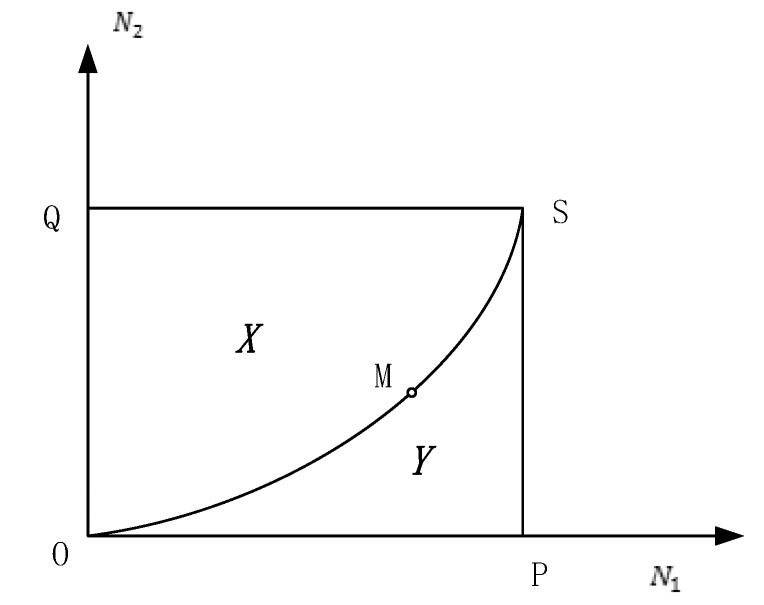
Phase diagram of the two-dimensional system.

Figure 5 shows the phase diagram of the two-dimensional system of *N*_1_ and *N*_2_:

When $\left\{ \begin{array}{l} r_{1}r_{2}>mn\\ r_{1}>-m\\ r_{2}>-n \end{array}\right.$ or $\left\{ \begin{array}{l} r_{1}r_{2}<mn\\ r_{1}<-m\\ r_{2}<-n \end{array}\right.$, *M* is the saddle point. $\overset{⌢}{OM}$ and $\overset{⌢}{MS}$ are the dividing lines and both tend to *M*, dividing the area into *X* and *Y*.

#### 4.1.2. Four-Dimensional Equilibrium Point

The equation shown is Equation (A5). The equilibrium points $P_{0}{\left(0,0,0,0\right)}^{T}$, $P_{1}{\left(K_{1},0,0,0\right)}^{T}$ and $P_{3}{\left(0,0,K_{2},0\right)}^{T}$ are persistent in area *G*^’^: $G^{’}=\left\{{\left(N_{1},I_{1},N_{2},I_{2}\right)}^{T}|K_{i}\geq N_{i}\geq I_{i}\geq 0,\;i=1,2\right\}$. Since the two-dimensional system is included in the four-dimensional one, the equilibrium solution of the two-dimensional system is used to solve the four-dimensional system’s. According to the definition of the threshold ($R_{0}=\frac{\beta N}{\gamma }$) in the infectious disease model, we let $R_{1}=\frac{\beta_{11}K_{1}}{\gamma_{1}}\;\mathrm{and}\;R_{2}=\frac{\beta_{22}K_{2}}{\gamma_{2}}$.

1. When *N*_1_ = *K*_1_, *N*_2_ = 0, since $0\leq I_{2}\leq N_{2}$, *I*_2_ = 0. According to Equation (A6), $\beta_{11}I_{1}\left(K_{1}-I_{1}\right)-\gamma_{1}I_{1}=0$. When *I*_1_ = 0, it coincides with point $P_{1}{\left(K_{1},0,0,0\right)}^{T}$, then $I_{1}=\frac{\beta_{11}K_{1}-\gamma_{1}}{\beta_{11}}$; Since $I_{1}=\frac{\beta_{11}K_{1}-\gamma_{1}}{\beta_{11}}>0$, $\beta_{11}K_{1}-\gamma_{1}>0$. That is, when $R_{1}=\frac{\beta_{11}K_{1}}{\gamma_{1}}>1$, there is an equilibrium point $P_{2}{\left(K_{1},I_{1E},0,0\right)}^{T}$, in which $I_{1E}=\frac{\beta_{11}K_{1}-\gamma_{1}}{\beta_{11}}=K_{1}\left(1-\frac{\gamma_{1}}{\beta_{11}K_{1}}\right)=K_{1}\left(1-\frac{1}{R_{1}}\right)$
2. When *N*_1_ = 0, *N*_2_ = *K*_2_ and $R_{2}=\frac{\beta_{22}K_{2}}{\gamma_{2}}>0$, there is equilibrium point $P_{4}{\left(0,0,K_{2},I_{2E}\right)}^{T}$, in which $I_{2E}=\frac{\beta_{22}K_{2}-\gamma_{2}}{\beta_{22}}=K_{1}\left(1-\frac{1}{R_{2}}\right)$
3. When $N_{1}=N_{1E}=\frac{\left(m+r_{1}\right)r_{2}K_{1}}{r_{1}r_{2}-mn}$, $N_{2}=N_{2E}=\frac{\left(n+r_{2}\right)r_{1}K_{2}}{r_{1}r_{2}-mn}$. According to Equation (A5), both *m* and *n* are smaller than 0, and Equation (A6) in the Appendix A is obtained. When *I*_1_ = 0 and *I*_2_ = 0, there is an equilibrium point $P_{5}{\left(N_{1E},0,N_{2E},0\right)}^{T}$, which satisfies $\left\{ \begin{array}{l} r_{1}r_{2}>mn\\ r_{1}>-m\\ r_{2}>-n \end{array}\right.$ or $\left\{ \begin{array}{l} r_{1}r_{2}<mn\\ r_{1}<-m\\ r_{2}<-n \end{array}\right.$
4. When $I_{1}I_{2}\neq 0$, there is an equilibrium point $P_{6}{\left(N_{1E},N_{1EE},N_{2E},N_{2EE}\right)}^{T}$, which satisfies $\left\{ \begin{array}{l} r_{1}r_{2}>mn\\ r_{1}>-m\\ r_{2}>-n \end{array}\right.$ or $\left\{ \begin{array}{l} r_{1}r_{2}<mn\\ r_{1}<-m\\ r_{2}<-n \end{array}\right.$. We have $A-\beta_{11}N_{1E}>0$, $B-\beta_{22}N_{2E}>0$ and $\prod_{1}<\prod_{2}$, where $\prod_{1}=\frac{A-\beta_{11}N_{1E}}{\beta_{12}N_{1E}};\prod_{2}=\frac{\beta_{21}N_{2E}}{B-\beta_{22}N_{2E}}$; $A=\gamma_{1}-r_{1}\left(1-\frac{N_{1E}}{K_{1}}\right)-m\frac{N_{2E}}{K_{2}};B=\gamma_{2}-r_{2}\left(1-\frac{N_{2E}}{K_{2}}\right)-n\frac{N_{1E}}{K_{1}}$.

Thus, the following equilibrium points are obtained in different regions: $P_{0}{\left(0,0,0,0\right)}^{T}$, $P_{1}{\left(K_{1},0,0,0\right)}^{T}$, $P_{2}{\left(K_{1},I_{1E},0,0\right)}^{T}$, $P_{3}{\left(0,0,K_{2},0\right)}^{T}$, $P_{4}{\left(0,0,K_{2},I_{2E}\right)}^{T}$, $P_{5}{\left(N_{1E},0,N_{2E},0\right)}^{T}$ and $P_{6}{\left(N_{1E},N_{1EE},N_{2E},N_{2EE}\right)}^{T}$. The stability of each equilibrium point in Region $G^{’}$ is discussed below.

### 4.2. Stability Analysis

#### 4.2.1. Stability of Equilibrium Points

Following [20], we use the Lyapunov Method and the Jacobi Matrix to explore the existence conditions and stability of the equilibrium point of the model. Table 3 lists the stability of equilibrium points under different conditions.

The meaning of the symbols in the table:


$\Omega_{1}=G^{’}=\left\{{\left(N_{1},I_{1},N_{2},I_{2}\right)}^{T}|K_{i}\geq N_{i}\geq I_{i}\geq 0,\;i=1,2\right\}$

$\Omega_{2}=\left\{{\left(N_{1},I_{1},N_{2},I_{2}\right)}^{T}\in G^{’}|{\left(N_{1},N_{2}\right)}^{T}\in Y\right\}$

$\Omega_{3}=\left\{{\left(N_{1},I_{1},N_{2},I_{2}\right)}^{T}\in G^{’}|{\left(N_{1},N_{2}\right)}^{T}\in Y,\;I_{1}\neq 0\right\}$

$\Omega_{4}=\left\{{\left(N_{1},I_{1},N_{2},I_{2}\right)}^{T}\in G^{’}|N_{1}\neq 0\right\}$

$\Omega_{5}=\left\{{\left(N_{1},I_{1},N_{2},I_{2}\right)}^{T}\in G^{’}|N_{1}\neq 0,\;I_{1}\neq 0\right\}$

$\Omega_{6}=\left\{{\left(N_{1},I_{1},N_{2},I_{2}\right)}^{T}\in G^{’}|{\left(N_{1},N_{2}\right)}^{T}\in X\right\}$

$\Omega_{7}=\left\{{\left(N_{1},I_{1},N_{2},I_{2}\right)}^{T}\in G^{’}|{\left(N_{1},N_{2}\right)}^{T}\in X,\;I_{2}\neq 0\right\}$

$\Omega_{8}=\left\{{\left(N_{1},I_{1},N_{2},I_{2}\right)}^{T}\in G^{’}|N_{2}\neq 0\right\}$

$\Omega_{9}=\left\{{\left(N_{1},I_{1},N_{2},I_{2}\right)}^{T}\in G^{’}|N_{2}\neq 0,\;I_{2}\neq 0\right\}$

$\Omega_{10}=\left\{{\left(N_{1},I_{1},N_{2},I_{2}\right)}^{T}\in G^{’}|N_{1}\neq 0,\;N_{2}\neq 0\right\}$

$\Omega_{11}=\left\{{\left(N_{1},I_{1},N_{2},I_{2}\right)}^{T}\in G^{’}|\begin{array}{l} N_{1}\neq 0,\;I_{1}\neq 0,\\ N_{2}\neq 0,\;I_{2}\neq 0 \end{array}\right\}$

$\Omega_{12}=\left\{{\left(N_{1},I_{1},N_{2},I_{2}\right)}^{T}\in G^{’}|{\left(N_{1},N_{2}\right)}^{T}\in \overset{⌢}{OM}\cup \overset{⌢}{MS}\right\}$

$\Omega_{13}=\left\{{\left(N_{1},I_{1},N_{2},I_{2}\right)}^{T}\in G^{’}|{\left(N_{1},N_{2}\right)}^{T}\in \overset{⌢}{OM}\cup \overset{⌢}{MS},\;I_{1}\neq 0,\;I_{2}\neq 0\right\}.$



*X* and *Y* are regions composed of *N*_1_ and *N*_2_ in the two-dimensional system.

#### 4.2.2. Stability of the Dual-Channel Pharmaceutical Supply Chain

Table 4 shows that when $R_{1}>1$ ($\beta_{11}$ is larger, $\gamma_{1}$ is smaller), offline sales remain at a stable level, while online sales are poor, and the total sales of the company tended to be offline sales. When $R_{2}>1$ ($\beta_{22}$ is larger, $\gamma_{2}$ is smaller), then online sales remain stable and offline sales are poor, and the total sales tend to be online sales. When $B-\beta_{22}N_{2E}>0$, $\prod_{1}=\frac{A-\beta_{11}N_{1E}}{\beta_{12}N_{1E}}<\prod_{2}=\frac{\beta_{21}N_{2E}}{B-\beta_{22}N_{2E}}$, $\beta_{11}$, $\beta_{12}$, $\beta_{21}$, $\beta_{22}$ are larger, both online and offline sales are at a stable level, and total dual-channel sales tend to be stable.

## 5. Simulation Analysis

This paper mainly discusses the change of the total sales volume of pharmaceuticals in the dual channels, and explores the possible coordination. When $P_{0}$, $P_{1}$, $P_{3}$, $P_{5}$ are in an unstable condition, the sales volume tends to zero, a situation that companies would wish to avoid. Simulation analysis is used to analyze the coordination of the dual channel pharmaceutical supply chain.. Due to the instability of $P_{0}$, it will not be discussed further. A positive balance only occurs when there is competition between the two channels. 

### 5.1. Analyses under a Stable $P_{1}{\left(K_{1},0,0,0\right)}^{T}$

$R_{1}=\frac{\beta_{11}K_{1}}{\gamma_{1}}<1$ exists under the two circumstances of $P_{1}{\left(K_{1},0,0,0\right)}^{T}$. When it meets the requirement that $R_{1}=\frac{\beta_{11}K_{1}}{\gamma_{1}}>1$, $P_{1}$ can be converted to $P_{2}$. This means if the $\beta_{11}$ increases and $\gamma_{1}$ decreases, the offline sales volume improve and the total sales volume of the enterprise improves as well. Assume that $\gamma_{1}=\gamma_{2}=1$, $K_{1}=K_{2}=1000$, $N_{1}\left(0\right)=N_{2}\left(0\right)=100,\;m=-1.2,\;n=-1.5,\;t=300,$ $\gamma_{1}=\gamma_{2}=0.01$, $\beta_{12}=0.05,\beta_{22}=0.05,\;\beta_{21}=0.05,$ $\beta_{11}=0.000005;0.00003;0.00006;0.00009,$ it meets the requirement that $R_{1}=\frac{\beta_{11}K_{1}}{\gamma_{1}}=0.5<1$.

**Figure 6:**
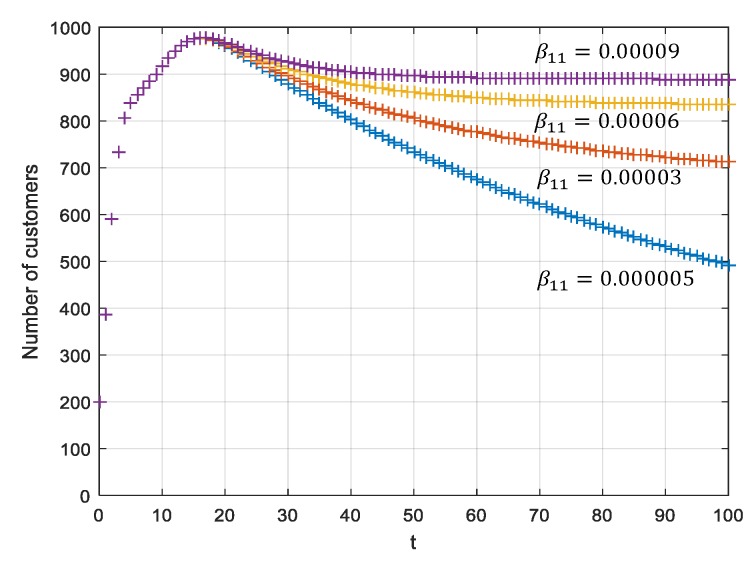
Total sales volumes for the dual-channel supply chain for different $\beta_{11}$.

As shown in Figure 6, it presents a change in the total sales volume of dual-channel pharmaceutical companies at time t. The total sales volume of the dual channel increases with the increase of the offline transmission influence rate $\beta_{11}$, achieving the coordination of the two channels.

### 5.2. Analyses under a Stabile of $P_{3}{\left(0,0,K_{2},0\right)}^{T}$

When it meets the requirement that $R_{2}=\frac{\beta_{22}K_{2}}{\gamma_{2}}>1$, $P_{3}{\left(0,0,K_{2},0\right)}^{T}$ can be converted to $P_{4}{\left(0,0,K_{2},I_{2E}\right)}^{T}$. This means that if the $\beta_{22}$ increases and $\gamma_{2}$ decreases, online sales volumes improve, as do the total sales volumes of the enterprise. Assume that $\gamma_{1}=\gamma_{2}=1$, $K_{1}=K_{2}=1000$, $N_{1}\left(0\right)=N_{2}\left(0\right)=100,\;m=-1.2,\;n=-0.5,\;t=10,$ $\gamma_{1}=\gamma_{2}=0.01$, $\beta_{11}=0.00005$, $\beta_{12}=0.00005$ $\beta_{22}=0.000005;0.00003;0.00006;0.00009$ and $\beta_{21}=0.00005,$

**Figure 7:**
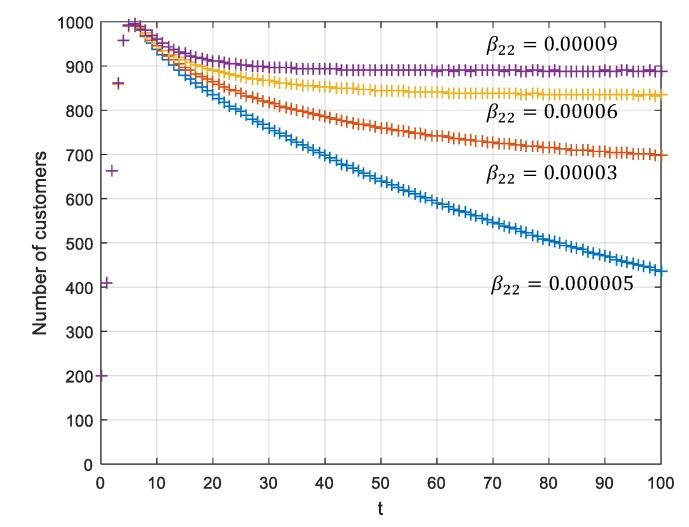
Total sales volumes of the dual-channel supply chain for different $\beta_{22}$.

As shown in Figure 7, the total sales volume of the dual-channel increases with the increase of the online transmission influence rate $\beta_{22}$, achieving the coordination of the two channels.

### 5.3. Analyses under a Stabile $P_{5}{\left(N_{1E},0,N_{2E},0\right)}^{T}$

**Figure 8:**
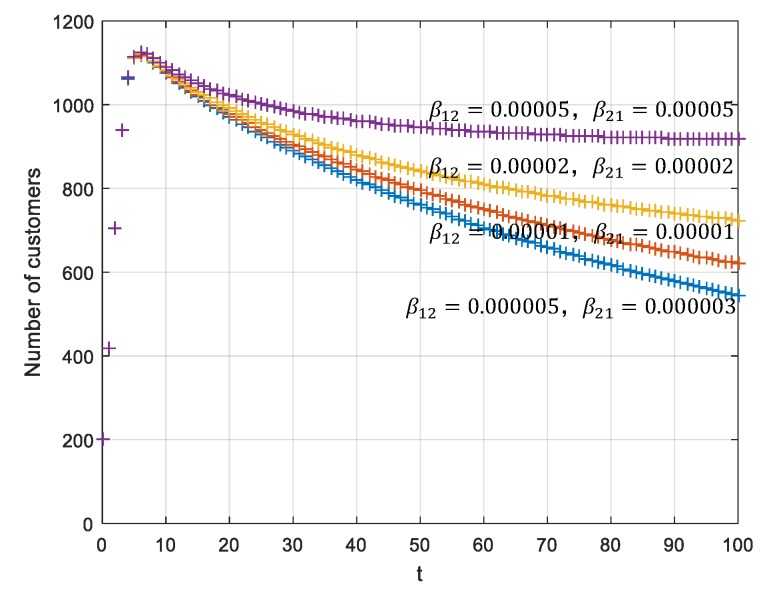
Total sales volumes of the dual-channel supply chain.

When it meets the requirement $\prod_{1}=\frac{A-\beta_{11}N_{1E}}{\beta_{12}N_{1E}}<\prod_{2}=\frac{\beta_{21}N_{2E}}{B-\beta_{22}N_{2E}}$, $P_{5}{\left(N_{1E},0,N_{2E},0\right)}^{T}$ can be converted to $P_{6}{\left(N_{1E},N_{1EE},N_{2E},N_{2EE}\right)}^{T}$. This means that if the $\beta_{12}$ and $\beta_{21}$ increase, both offline and online sales volumes improve and the total sales volume of the enterprise improves as well. Assume that $\gamma_{1}=\gamma_{2}=1$, $K_{1}=K_{2}=1000$, $N_{1}\left(0\right)=N_{2}\left(0\right)=100,\;m=-0.8,\;n=-0.5,\;t=10,$ $\gamma_{1}=\gamma_{2}=0.01$, $\beta_{11}=0.00002$, $\beta_{22}=0.00001$, $\beta_{12}=0.000005;0.00001;0.00002;0.00005$, and $\beta_{22}=0.000003;0.00001;0.00002;0.00005$,

As shown in Figure 8, the total sales volume of the dual channels increases with the increase of the offline and online transmission influence rate $\beta_{12}$ and $\beta_{21}$. Thus, dual channel coordination is achieved.

## 6. Conclusions

This paper first discussed the offline and online channels of a pharmaceutical supply chain. It then constructed the susceptible-infected-susceptible epidemic model for the dual-channel supply chain. Finally, it derived the equilibrium points and conducted simulation analyses. Our results show that the increase in offline transmission influence rate $\beta_{11}$, online transmission influence rate $\beta_{22}$, offline transmission influence rate $\beta_{12}$, and offline transmission influence rate $\beta_{21}$ can improve the total sales in the pharmaceutical supply chain. Based on our results, the following managerial insights are obtained:

1. Offline channels can reduce prices, enhance service quality, and attract consumers who prefer offline channels to become buyers. Enterprises can use revenue-sharing supply chain contracts and other methods to obtain lower wholesale prices and lower pharmaceutical prices, thereby achieving sales growth. They can also provide comprehensive pre-sales and after-sales services.
2. Online channels can provide online consultation and diversified distribution services. Professionals can provide consulting services for online consumers. For customers with time constraints, pharmaceutical enterprises can cooperate with express logistics enterprises. For more price-sensitive customers, as long as the reliability of shipping is guaranteed, they would choose cheaper modes of transportation.
3. Online and offline channels should differentiate between their target customers. The online channel should target young customers, who have a higher acceptance of new things. In contrast, offline channels should target older people as customers as they are more willing to visit an offline store. Online and offline channels should also differentiate brands and packaging, and control the price gap within a reasonable range.
4. Dual-channel pharmaceutical supply chains need to achieve the mutual coordination of online and offline flow and information sharing. Through remote diagnosis and treatment and O2O in certain regions, we can improve the information sharing of offline and online channels, including marketing strategies, market information, information distribution, and so on. In this way, dual-channel pharmaceutical supply chains can realize the coordination of marketing and logistics.

There are several limitations in this research. Especially, the potential factors in the dual-channel SIS model are not considered comprehensively. There are still many potential problems to be further explored. First, future research can conduct more detailed quantitative analysis on the factor of the influence rate. Second, actual data can be collected based on which our models and results can be tested empirically. Third, issues like network design, channel selection, and fulfillment service contracts can be addressed for dual-channel pharmaceutical supply chains [26,27].

## Figures and Tables

**Table 1:** Offline pharmaceutical supply chain structure.

	Offline Channel	Structure
B2B	Agency Model	Pharmaceutical manufacturers sell pharmaceuticals through agents or distributors
	Direct Model	Pharmaceutical manufacturers directly sell pharmaceuticals to medical institutions and pharmacies without agents or distributors
B2C	Hospital Channel	Patients purchase medicine from hospital pharmacy after a doctor prescribes prescription
	Pharmacy Channel	Consumers directly purchase OTC pharmaceuticals, medical devices, health care products, etc., or purchase prescription pharmaceuticals according to prescriptions in the pharmacy

Source: Summarized according to references.

**Table 2:** Online pharmaceutical supply chain structure.

	Online Channel	Structure
B2B	Self-operated	Enterprises build online platforms for pharmaceutical sales to other enterprises
	Third-party platform	E-commerce platform build by third-party for business to business transation
B2C	Self-operated	Enterprises build online platforms for pharmaceutical sales to customers
	Third-party Platform	E-commerce platform build by third-party for business to consumer transation
O2O	Self-operated	Pharmaceutical retailer B2C + Online information release + offline pharmaceutical delivery and payment
	Third-party platform	Online ordering and offline delivery established by third-party platforms

Source: Summarized according to references.

**Table 3:** Stability of equilibrium point of the four-dimensional system.

Existence Condition			Stability of Equilibrium Points
			$P_{0}$ is unstable in $\Omega_{1}$
$\left\{ \begin{array}{l} r_{1}r_{2}<mn\\ r_{1}<-m\\ r_{2}<-n \end{array}\right.$	$R_{1}<1$		$P_{1}$ is asymptotically stable in $\Omega_{2}$
	$R_{1}>1$		$P_{2}$ exists, asymptotically stable in $\Omega_{3}$
	$R_{2}<1$		$P_{3}$ is asymptotically stable in $\Omega_{6}$
	$R_{2}>1$		$P_{4}$ exists, asymptotically stable in $\Omega_{7}$
	$A-\beta_{11}N_{1E}>0$ $B-\beta_{22}N_{2E}>0$	$\prod_{1}>\prod_{2}$	$P_{5}$ exists, asymptotically stable in $\Omega_{12}$
		$\prod_{1}<\prod_{2}$	$P_{6}$ exists, asymptotically stable in $\Omega_{13}$
$\left\{ \begin{array}{l} r_{1}r_{2}>mn\\ r_{1}>-m\\ r_{2}>-n \end{array}\right.$	$A-\beta_{11}N_{1E}>0$ $B-\beta_{22}N_{2E}>0$	$\prod_{1}>\prod_{2}$	$P_{5}$ exists, asymptotically stable in $\Omega_{10}$
		$\prod_{1}<\prod_{2}$	$P_{6}$ exists, asymptotically stable in $\Omega_{11}$
$\left\{ \begin{array}{l} r_{1}r_{2}>mn\\ r_{2}<-n \end{array}\right.\;\mathrm{or}\;\left\{ \begin{array}{l} r_{1}r_{2}<mn\\ r_{1}>-m \end{array}\right.$	$R_{1}<1$		$P_{1}$ is asymptotically stable in $\Omega_{4}$
	$R_{1}>1$		$P_{2}$ exists, asymptotically stable in $\Omega_{5}$
$\left\{ \begin{array}{l} r_{1}r_{2}<mn\\ r_{2}>-n \end{array}\right.\;\mathrm{or}\;\left\{ \begin{array}{l} r_{1}r_{2}>mn\\ r_{1}<-m \end{array}\right.$	$R_{2}<1$		$P_{3}$ is asymptotically stable in $\Omega_{8}$
	$R_{2}>1$		$P_{4}$ exists, asymptotically stable in $\Omega_{9}$

**Table 4:** Stability of the dual-channel pharmaceutical supply chain.

Existence Condition		Offline	Online		Total Sales	
		Demand	Sales	Demand	Sales	
$\mathrm{No}\;\mathrm{Constraint}\;\mathrm{in}\;\mathrm{Area}\;G^{\prime }$		Does Not Approach 0	Do Not Approach 0	Does Not Approach 0	Do Not Approach 0	Do Not Approach 0
$\left\{ \begin{array}{l} r_{1}r_{2}<mn\\ r_{1}<-m\\ r_{2}<-n \end{array}\right.m,n<0$	$R_{1}=\frac{\beta_{11}K_{1}}{\gamma_{1}}<1$; $\beta_{11}$ smaller, $\gamma_{1}$ larger; in region $\Omega_{2}$	*K*_1_ *a*	0	0	0	0
	$R_{1}=\frac{\beta_{11}K_{1}}{\gamma_{1}}>1$; $\beta_{11}$ larger, $\gamma_{1}$ smaller; in region $\Omega_{3}$	*K*_1_	*I*_1*E*_	0	0	*I*_1*E*_
	$R_{2}=\frac{\beta_{22}K_{2}}{\gamma_{2}}<1$; $\beta_{22}$ smaller, $\gamma_{2}$ larger; in region $\Omega_{6}$	0	0	*K*_2_	0	0
	$R_{2}=\frac{\beta_{22}K_{2}}{\gamma_{2}}>1$; $\beta_{22}$ larger, $\gamma_{2}$ smaller; in region $\Omega_{7}$	0	0	*K*_2_	*I*_2*E*_	*I*_2*E*_
	$\beta_{11}$, $\beta_{12}$, $\beta_{21}$, $\beta_{22}$ smaller; in region $\Omega_{12}$	*N*_1*E*_	0	*N*_2*E*_	0	0
	$\beta_{11}$, $\beta_{12}$, $\beta_{21}$, $\beta_{22}$ larger; in region $\Omega_{13}$	*N*_1*E*_	*I*_1*EE*_	*N*_2*E*_	*I*_2*EE*_	*I*_1*EE*_ + *I*_2*EE*_
$\left\{ \begin{array}{l} r_{1}r_{2}>mn\\ r_{1}>-m\\ r_{2}>-n \end{array}\right.m,n<0$	$\beta_{11}$, $\beta_{12}$, $\beta_{21}$, $\beta_{22}$ smaller; in region $\Omega_{10}$	*N*_1*E*_	0	*N*_2*E*_	0	0
	$\beta_{11}$, $\beta_{12}$, $\beta_{21}$, $\beta_{22}$ larger; in region $\Omega_{11}$	*N*_1*E*_	*I*_1*EE*_	*N*_2*E*_	*I*_2*EE*_	*I*_1*EE*_ + *I*_2*EE*_
$\left\{ \begin{array}{l} r_{1}r_{2}>mn\\ r_{2}<-n \end{array}\right.\;\mathrm{or}\;\left\{ \begin{array}{l} r_{1}r_{2}<mn\\ r_{1}>-m \end{array}\right.$(Unable to judge the positive and negative of *m* and *n*)	$R_{1}=\frac{\beta_{11}K_{1}}{\gamma_{1}}<1$; $\beta_{11}$ smaller, $\gamma_{1}\;\mathrm{larger};\;\mathrm{in}\;\mathrm{region}$	*K*_1_	0	0	0	0
	$R_{1}=\frac{\beta_{11}K_{1}}{\gamma_{1}}>1$; $\beta_{11}$ smaller, $\gamma_{1}$ larger; in region $\Omega_{5}$	*K*_1_	*I*_1*E*_	0	0	*I*_1*E*_
$\left\{ \begin{array}{l} r_{1}r_{2}<mn\\ r_{2}>-n \end{array}\right.\;\mathrm{or}\;\left\{ \begin{array}{l} r_{1}r_{2}>mn\\ r_{1}<-m \end{array}\right.$(Unable to judge the positive and negative of *m* and *n*)	$R_{2}=\frac{\beta_{22}K_{2}}{\gamma_{2}}<1$; $\beta_{22}$ smaller, $\gamma_{2}$ larger; in region $\Omega_{8}$	0	0	*K*_2_	0	0
	$R_{2}=\frac{\beta_{22}K_{2}}{\gamma_{2}}>1$; $\beta_{22}$ smaller, $\gamma_{2}$ larger; in region $\Omega_{9}$	0	0	*K*_2_	*I*_2*E*_	*I*_2*E*_

## Appendix A

(A1) $$\left\{ \begin{array}{l} \frac{dN_{1}}{dt}=\left[r_{1}\left(1-\frac{N_{1}}{K_{1}}\right)+m\frac{N_{2}}{K_{2}}\right]N_{1}\\ \frac{dS_{1}}{dt}=r_{1}\left(1-\frac{N_{1}}{K_{1}}+m\frac{N_{2}}{K_{2}}\right)S_{1}-\\ \left(\beta_{11}I_{1}+\beta_{12}I_{2}\right)S_{1}+\gamma_{1}I_{1}\\ \frac{dI_{1}}{dt}=r_{1}\left(1-\frac{N_{1}}{K_{1}}+m\frac{N_{2}}{K_{2}}\right)I_{1}+\\ \left(\beta_{11}I_{1}+\beta_{12}I_{2}\right)S_{1}-\gamma_{1}I_{1}\\ \frac{dN_{2}}{dt}=\left[r_{2}\left(1-\frac{N_{2}}{K_{2}}\right)+n\frac{N_{1}}{K_{1}}\right]N_{2}\\ \frac{dS_{2}}{dt}=r_{2}\left(1-\frac{N_{2}}{K_{2}}+n\frac{N_{1}}{K_{1}}\right)S_{2}-\\ \left(\beta_{21}I_{1}+\beta_{22}I_{2}\right)S_{2}+\gamma_{2}I_{2}\\ \frac{dI_{2}}{dt}=r_{2}\left(1-\frac{N_{2}}{K_{2}}+n\frac{N_{1}}{K_{1}}\right)I_{2}+\\ \left(\beta_{21}I_{1}+\beta_{22}I_{2}\right)S_{2}-\gamma_{2}I_{2}\\ \frac{dI}{dt}=\frac{dI_{1}}{dt}+\frac{dI_{2}}{dt}=r_{1}\left(1-\frac{N_{1}}{K_{1}}+m\frac{N_{2}}{K_{2}}\right)I_{1}+\\ \left(\beta_{11}I_{1}+\beta_{12}I_{2}\right)S_{1}-\gamma_{1}I_{1}+\\ r_{2}\left(1-\frac{N_{2}}{K_{2}}+n\frac{N_{1}}{K_{1}}\right)I_{2}+\left(\beta_{21}I_{1}+\beta_{22}I_{2}\right)S_{2}-\gamma_{2}I_{2}\\ 0\leq N_{i}=S_{i}+I_{i}\leq K_{i},\;i=1\;\mathrm{or}\;2;\;r_{i}>0,\;i=1\;\mathrm{or}\;2 \end{array}\right.$$

where the notations are defined as below:

*K*_1_, *K*_2_: Total market sales of the offline and online channels for a pharmaceutical product, respectively.

*S*_1_, *S*_2_: The number of customers who prefer offline (online) channels, representing offline (online) total demand;

*I*_1_, *I*_2_: The number of customers who purchased pharmaceuticals in the offline (online) channel of a pharmaceutical company, representing the sales volume of offline (online) channels; these customers have influence on the purchase channel choices of customers who haven’t purchased pharmaceuticals;

*I*: total online and offline sales;

*N*_1_, *N*_2_: The sum of customers of a pharmaceutical company who have a purchase preference for offline (online) channel and those who have purchased through offline (online) channels;

*r*_1_, *r*_2_: The intrinsic growth rate of customers in the offline (online) channel which is the customer growth rate under natural channel optimization and environmental impact;

$r_{1}\left(1-\frac{N_{1}}{K_{1}}\right)N_{1}$, $r_{2}\left(1-\frac{N_{2}}{K_{2}}\right)N_{2}$: Natural growth of offline (online) channels;

*m*: The coefficient of influence of total online channel demand on the total offline channel demand;

*n*: The coefficient of influence of total offline channel demand on the total online channel demand; When m and *n* are both positive, it means that the two channels are mutually beneficial. When *m* and *n* are both negative, it means that the two channels are in a competitive state. When one is positive and one negative, the two channels are parasitic;

β: The impact of purchased customers on potential customers; Following [9], *β* is regarded as a coordination coefficient of corporate channel conflicts under the dual-channel environment of pharmaceutical companies;

$\beta_{11}$, $\beta_{22}$: Impact of purchased customers in offline (online) channels on customers with offline (online) channel preferences;

$\beta_{12}$, $\beta_{21}$: Impact of purchased customers in offline (online) channels on customer with online (offline) channel preferences;

$\gamma_{1}$, $\gamma_{2}$: The restore rate of offline (online) channels, which refers to the return rate of offline (online) channels within a given time.

(A2) $$\left\{ \begin{array}{l} \frac{dN_{1}}{dt}=\left[r_{1}\left(1-\frac{N_{1}}{K_{1}}\right)+m\frac{N_{2}}{K_{2}}\right]N_{1}\\ \frac{dI_{1}}{dt}=r_{1}\left(1-\frac{N_{1}}{K_{1}}\right)I_{1}+\left(\beta_{11}I_{1}+\beta_{12}I_{2}\right)\left(N_{1}-I_{1}\right)\\ +m\frac{N_{2}}{K_{2}}I_{1}-\gamma_{1}I_{1}\\ \frac{dN_{2}}{dt}=\left[r_{2}\left(1-\frac{N_{2}}{K_{2}}\right)+n\frac{N_{1}}{K_{1}}\right]N_{2}\\ \frac{dI_{2}}{dt}=r_{2}\left(1-\frac{N_{2}}{K_{2}}\right)I_{2}+\left(\beta_{21}I_{1}+\beta_{22}I_{2}\right)\left(N_{2}-I_{2}\right)\\ +n\frac{N_{1}}{K_{1}}I_{2}-\gamma_{2}I_{2}\\ K_{i}\geq N_{i}\geq I_{i}\geq 0,\;i=1\;\mathrm{or}\;2\\ r_{i}>0,\;i=1\;\mathrm{or}\;2 \end{array}\right.$$

(A3) $$\left\{ \begin{array}{l} \frac{dN_{1}}{dt}=\left[r_{1}\left(1-\frac{N_{1}}{K_{1}}\right)+m\frac{N_{2}}{K_{2}}\right]N_{1}\\ \frac{dN_{2}}{dt}=\left[r_{2}\left(1-\frac{N_{2}}{K_{2}}\right)+n\frac{N_{1}}{K_{1}}\right]N_{2}\\ K_{i}\geq N_{i}\geq 0,\;i=1\;\mathrm{or}\;2\\ r_{i}>0,\;i=1\;\mathrm{or}\;2 \end{array}\right.$$

(A4) $$\left\{ \begin{array}{l} r_{1}\left(1-\frac{N_{1}}{K_{1}}\right)+m\frac{N_{2}}{K_{2}}=0\\ r_{2}\left(1-\frac{N_{2}}{K_{2}}\right)+n\frac{N_{1}}{K_{1}}=0\\ K_{i}\geq N_{i}\geq 0,\;i=1\;\mathrm{or}\;2\\ r_{i}>0,\;i=1\;\mathrm{or}\;2 \end{array}\right.$$

(A5) $$\left\{ \begin{array}{l} \frac{dN_{1}}{dt}=\left[r_{1}\left(1-\frac{N_{1}}{K_{1}}\right)+m\frac{N_{2}}{K_{2}}\right]N_{1}=0\\ \frac{dI_{1}}{dt}=r_{1}\left(1-\frac{N_{1}}{K_{1}}\right)I_{1}+\left(\beta_{11}I_{1}+\beta_{12}I_{2}\right)\left(N_{1}-I_{1}\right)\\ +m\frac{N_{2}}{K_{2}}I_{1}-\gamma_{1}I_{1}=0\\ \frac{dN_{2}}{dt}=\left[r_{2}\left(1-\frac{N_{2}}{K_{2}}\right)+n\frac{N_{1}}{K_{1}}\right]N_{2}=0\\ \frac{dI_{2}}{dt}=r_{2}\left(1-\frac{N_{2}}{K_{2}}\right)I_{2}+\left(\beta_{21}I_{1}+\beta_{22}I_{2}\right)\left(N_{2}-I_{2}\right)\\ +n\frac{N_{1}}{K_{1}}I_{2}-\gamma_{2}I_{2}=0\\ K_{i}\geq N_{i}\geq I_{i}\geq 0,\;i=1\;\mathrm{or}\;2\\ r_{i}>0,\;i=1\;\mathrm{or}\;2 \end{array}\right.$$

(A6) $$\left\{ \begin{array}{l} r_{1}\left(1-\frac{N_{1E}}{K_{1}}\right)I_{1}+\left(\beta_{11}I_{1}+\beta_{12}I_{2}\right)\left(N_{1E}-I_{1}\right)\\ +m\frac{N_{2E}}{K_{2}}I_{1}-\gamma_{1}I_{1}=0\\ r_{2}\left(1-\frac{N_{2E}}{K_{2}}\right)I_{2}+\left(\beta_{21}I_{1}+\beta_{22}I_{2}\right)\left(N_{2E}-I_{2}\right)\\ +n\frac{N_{1E}}{K_{1}}I_{2}-\gamma_{2}I_{2}=0 \end{array}\right.$$
